# Delivery of antigen to porcine dendritic cells by fusing antigen with porcine dendritic cells targeting peptide

**DOI:** 10.3389/fimmu.2022.926279

**Published:** 2022-09-08

**Authors:** Tian Xia, Ning Wang, Yuqing Tang, Yueyi Gao, Chong Gao, Jianhui Hao, Yanping Jiang, Xiaona Wang, Zhifu Shan, Jiaxuan Li, Han Zhou, Wen Cui, Xinyuan Qiao, Lijie Tang, Li Wang, Yijing Li

**Affiliations:** ^1^ College of Veterinary Medicine, Northeast Agricultural University, Harbin, China; ^2^ Division of Viral Biologic Testing(I), China Institute of Veterinary Drug Control, Beijing, China; ^3^ China Ministry of Agriculture Key Laboratory of Animal Pathogen Biology, Northeastern Science Inspection Station, Harbin, China

**Keywords:** porcine dendritic cells, CTLA4, targeting, recombinant lactobacillus, fusion expression

## Abstract

Dendritic cells (DCs) are professional antigen-presenting cells that can recognize, capture, and process antigens. Fusing molecules targeting DCs with antigens can effectively improve the efficiency with which antigens are recognized and captured by DCs. This targeting strategy can be used for vaccine development to effectively improve the efficiency of antigen recognition and capture by DCs. The targeting sequence of porcine cytotoxic T-lymphocyte associated protein 4 (CTLA4), which binds porcine DCs, was identified in this study. Recombinant *Lactobacillus reuteri (L. reuteri)* expressing CTLA4-6aa (LYPPPY) and CTLA4-87aa fused to the porcine epidemic diarrhea virus (PEDV) protective antigen core neutralizing epitope (COE) were used to evaluate the ability of the two targeting motifs to bind the B7 molecule on DCs. Our results demonstrate that CTLA4-6aa could bind porcine DCs, and recombinant *Lactobacillus* expressing the CTLA4-6aa captured by porcine DCs was more efficient than those expressing CTLA4-87aa. In addition, the expression of DC markers, toll-like receptors, and cytokines was significantly higher in the 6aa-COE/*L. reuteri*-stimulated porcine DCs compared to DCs treated with 87aa-COE/*L. reuteri* (*p*<0.01) and recombinant *Lactobacillus* expressing CTLA4-6aa enhanced the ability of porcine DCs to activate T-cell proliferation. Our analysis of the protein structure revealed that CTLA4-87aa contains intramolecular hydrogen bonds, which may have weakened the intermolecular force between the residues on porcine CTLA4 and that on B7. In conclusion, recombinant *Lactobacillus* expressing CTLA4-6aa were more efficiently captured by porcine DCs and had a stronger ability to promote DC maturation and enhance T-cell proliferation. The LYPPPY motif is the optimal sequence for binding to porcine DCs. Piglets immunized with recombinant *Lactobacillus* showed that recombinant *Lactobacillus* expressing CTLA4-6aa induced significant levels of anti-PEDV-specific IgG and IgA antibody responses. Our study may promote research on DC-targeting strategies to enhance the effectiveness of porcine vaccines.

## 1 Introduction

Dendritic cells (DCs) are key regulators of T- and B-cell immunity. They can recognize, capture, process, and present antigens to T-cells, which play a critical role in the initiation and regulation of immune responses (1, 2). Moreover, DCs are the “bridge” cells that connect non-specific and specific immune responses and initiate immune responses (3). Consequently, DCs are usually targeted for the delivery of antigens (4). Using DC-targeting molecules enables the delivery of antigens to DCs specifically, which can improve the efficiency of antigens recognized and captured by these cells, leading to stronger T-cell responses (5, 6). Previous studies screened and identified human and chicken DC-targeting peptides using a phage display peptide library (7, 8). Subsequently, oral mucosal vaccines targeting DCs were proposed (9). However, inadequate research on porcine DC-targeting peptides has hampered the development of porcine vaccines.

DC surface receptors can bind specific ligands. Here, we propose a strategy that is based on the binding of natural ligands to the receptors on DCs (10). Increasing evidence indicates that specific ligands can facilitate the targeted delivery of antigens to DCs by interacting with the surface receptors on DCs (11, 12).

Moreover, screening and identifying the ligands that bind DC surface receptors and determining the key binding sites are effective strategies for confirming porcine DC peptides. Cytotoxic T-lymphocyte associated protein 4 (CTLA4) is mainly expressed on the surface of activated T-cells and binds to B7 on DCs (13, 14). CTLA4 consists of extracellular, transmembrane, and intracellular domains, and the extracellular domain plays an indispensable role in binding B7 (15). Many studies have used CTLA4 as a ligand for delivering antigens to DCs. Fusing the extracellular domain of CTLA4 with antigens can improve the efficiency with which antigens are recognized and captured by DCs (16–19). Peach et al. (1995) analyzed the amino acids in the extracellular domains of CTLA4 in humans, mice, cattle, sheep, dogs, pigs, cats, and rabbits and suggested that the extracellular domain of CTLA4 has a highly conserved MYPPPY motif (position 99-104, methionine (Met), Tyr, Pro, Pro, Pro, Tyr). Furthermore, the authors speculated that the conserved MYPPPY motif may be the main binding site in the extracellular domain of the molecule (20). However, the highly conserved MYPPPY motif, which is involved in B7 binding is not fully conserved in the porcine CTLA4 sequence; Met at position 99 is replaced by leucine (Leu) (21). In addition, Metzler et al. (1997) speculated that the regions flanking the MYPPPY motif may be equally important for binding DCs (22). Consequently, although it may be important to ascertain the role of the LYPPPY motif in porcine CTLA4 binding to porcine DCs, no formal studies have been performed to verify these speculations.

To explore the effects of antigen delivery with the help of DC-targeting molecules, we used *Lactobacillus reuteri* (*L. reuteri)* expressing antigens that were fused to DC-targeting molecules. *Lactobacillus* have become the most promising live bacterial vectors due to their probiotic effects (23, 24). *Lactobacillus*-live vector vaccines are prepared using genetic engineering, which enables the delivery of foreign antigens to the host mucosal system and supports the induction of a subsequent immune response (25, 26). *Lactobacillus*, serving as vectors that express and deliver antigens, play an important role in inducing intestinal mucosal immunity; however, several problems regarding their use as bacterial vectors remain unresolved. Compared with other expression vectors (such as E. coli, yeast, and insect expression vectors), *Lactobacillus* expression vectors produce lower amounts of antigen (27). An insufficient number of antigens makes it difficult to stimulate the body’s immune response. Thus, to enhance their immunogenicity and increase the efficiency with which *Lactobacillus* are recognized by DCs, it is common practice to include sequences of DC-targeting molecules in the recombinant *Lactobacillus* during vector construction. To date, several studies have analyzed and corroborated the feasibility of using a DC-targeted strategy for *Lactobacillus* vaccines (28–30). Accordingly, lactic acid bacteria have been widely employed as a delivery model to analyze the targeting functions of DC-targeting molecules.

Here, we aimed to elucidate the effectiveness of CTLA4-6aa (LYPPPY) as a DC-targeting peptide. Additionally, recombinant *Lactobacilli* expressing CTLA4-6aa and CTLA4-87aa fused to the porcine epidemic diarrhea virus (PEDV) protective antigen COE were used to evaluate the ability of the two targeting motifs to bind the B7 molecule on porcine DCs. Our study suggests that the targeting of DCs could be a potential strategy for the development of porcine vaccines.

## 2 Materials and methods

### 2.1 Bacteria and plasmids

Swine-derived *L. reuteri* was isolated from 3-month-old pig jejunum and cultured in de Man, Rogosa, and Sharpe medium (MRS; Hopebol, Qingdao, China) without shaking. The constitutive expression plasmid pPG-T7g10-PPT was constructed in our laboratory and contained the HCE strong constitutive promoter, T7g10 transcriptional enhancer, pgsA anchor from Bacillus subtilis for stabilizing the heterologous protein on the cell membrane (surface-displaying), and rrnBT1T2 terminator. The pMD19T-COE recombinant plasmid, recombinant plasmid that was inserted into the COE antigen was constructed and preserved in our laboratory. The core neutralizing epitope (COE) gene was obtained from the PEDV LJB/15 strain, which was isolated and identified from clinical samples in 2017 in our laboratory.

Animals

Nine antibody-seronegative, healthy One-month-old piglets were purchased from the Acheng Experimental Practice Base of Northeast Agricultural University (Harbin, China). Animal studies were performed according to the regulations of the Animal Experiment Ethics Committee of the Northeast Agricultural University, China (review number: NEAUEC20210337).

### 2.2 Peptide sequence

The LYPPPY motif (6aa) and the negative control peptide (YPLPYP, rearranging the sequence of 6aa) were synthesized by Jinsiri Biotechnology (Nanjing, Jiangsu) and purified by high-performance liquid chromatography (purity>95%). The N-terminus was labeled with fluorescein isothiocyanate (FITC).

### 2.3 Isolation and identification of porcine monocyte derived dendritic cells (MoDCs)

Blood collected from the precaval vein of 5–8-week-old swine was diluted with sterile phosphate-buffered saline (PBS), loaded onto an equal volume Histopaque-1077 (Sigma-Aldrich, St. Louis, MO, USA), and centrifuged at 800 × g for 25 min. The middle white mist-like cell layers were harvested and centrifuged at 600 × g for 15 min. The cell pellets were resuspended in red blood cell lysis buffer (Beyotime, Shanghai, China), rested for 5 min at 37°C, and thereafter centrifuged at 600 × g for 6 min. Cell pellets were washed twice with PBS and centrifuged at 600 × g for 6 min. Peripheral blood mononuclear cells (PBMCs) were obtained as mentioned above and seeded at 10^6^ cells mL^-1^ in 6-well plates containing pre-warmed Roswell Park Memorial Institute-1,640 (RPMI-1640) medium, and supplemented with 1 U ml^-1^ penicillin and streptomycin, and 10% fetal calf serum (Gibco, Grand Island, NY, USA) for 6 days at 37°C with 5% CO_2_. Half of the medium was replaced with pre-warmed complete medium every 2 days, and non-adherent cells were removed on day 2 and day 4. Approximately 20 ng mL^-1^ recombinant porcine granulocyte-macrophage colony-stimulating factor (GM-CSF) (BD Biosciences, San Jose, CA, USA) and 20 ng mL^-1^ interleukin (IL-4) (BD Biosciences, San Jose, CA, USA) were added to the medium. Morphological changes in immature porcine MoDCs were observed using optical microscopy.

Immature porcine MoDCs were harvested on day 7 and incubated with CD172a labeled by phycoerythrin (PE) and MHC II labeled by fluorescein isothiocyanate (FITC) for 30 min. The cells were washed three times with sterile PBS, resuspended in 500 µL sterile PBS, and analyzed using flow cytometry and fluorescence microscopy.

### 2.4 Determining the binding ability of the peptide

On day 6 of MoDC cultivation, complete medium was gently aspirated using pipettes and washed twice with PBS. Porcine MoDCs and intestinal epithelial cells (IPEC) were incubated with FITC-labeled 6aa peptide (LYPPPY) and FITC-labeled negative control peptide (YPLPYP) for 30 min at 4°C. After being washed three times with PBS, the MoDCs were fixed with 4% paraformaldehyde for 5 min at 37°C and observed under a fluorescence microscope (Bio-Rad, CA, USA). Meanwhile, MoDCs that were incubated with the two peptides were collected in Eppendorf tubes, resuspended in 500 µL sterile PBS, and subjected to flow cytometry (BD Biosciences, San Jose, CA, USA).

### 2.5 Construction of recombinant strains

Primers were designed according to the swine-derived CTLA4 genes, the complete coding sequence published in GenBank (Number NM214149.1), and synthesized by Comate Bioscience (Jilin, Co., Ltd.). The primers used are listed in **Table 1**. The 6aa-COE gene was amplified using forward and reverse primers (6aa-COE-F/6aa-COE-R) with the plasmid pMD19T-COE as the template. PCR amplification was performed as follows: 95°C for 5 min; 30 cycles at 98°C for 10 s, 55°C for 30 s, 68°C for 30 s, and 68°C for 10 min of final extension. The PCR products were confirmed by DNA sequencing. RNA was extracted from porcine lymph nodes, complementary DNA was obtained by reverse transcription, and the 87aa gene was amplified using forward and reverse primers (87aa-F/87aa-R) with complementary DNA as the template. PCR amplification was performed as follows: 95°C for 5 min; 30 cycles of 98°C for 10 s, 57°C for 15 s, 68°C for 30 s, and 68°C for 10 min of final extension. The PCR products were confirmed by DNA sequencing. Thereafter, the 6aa-COE and 87aa genes were cloned into the expression plasmid pPG-T7g10-PPT to generate pPG-6aa-COE and pPG-87aa-COE. The flag tag was inserted into the 6aa-COE fusion gene, and a recombinant expression plasmid was constructed as shown in **Figure S1**. To construct the recombinant *Lactobacillus* strain, recombinant plasmids were electrotransferred into *reuteri* and verified by PCR, double digestion, and sequencing.

**Table 1:** Primers sequences.

Plasmids	Primers	Sequence (5′ -3′)
87aa-COE	87aa-F	5′-CCCAAGCTTATGGTGTGTGAGTATG-3′
	87aa-R	5′-CCGCTCGAGCATACCCACATAGTAG-3′
6aa-COE	6aa-COE-F	5′-CGAGCTCATGCCCAAGCTT **CTGTACCCACCACCCTAC**CCGCTCGAG ***GAAGCCGCAGCCAAAGAG*** GCTGCAGCCAAGGTTACTTTGCCATCATT-3′
	6aa-COE-R	5′-GGGCCC ***CTTATCGTCGTCATCCTTGTAATC*** GTCCGTGACACCTTCAAGTGGTTTAGGCGTGCCAGT-3′

The restriction enzyme recognition sites used for cloning are underlined, the LYPPPY polypeptide sequence of porcine CTLA4 is shown in bold, the flag tag sequence is shown in bold and italic, and the rigid linker sequence with double underline is shown in bold and italic.

### 2.6 Protein expression and identification

Expression of the target proteins was analyzed by western blotting. The recombinant *Lactobacillus* were broken up by ultrasound and electrophoresis using 10% SDS-PAGE. Mouse anti-FLAG monoclonal antibody (diluted 1:1,000; Abcam, Cambridge, MA, USA) was used as the primary antibody, and horseradish peroxidase-conjugated goat anti-mouse IgG (diluted 1:5,000; ZSGB Biotech, Beijing, China) was used as the secondary antibody. Furthermore, 6aa-COE/*L. reuteri* and 87aa-COE/*L. reuteri* were cultured overnight at 37°C in MRS broth containing chloramphenicol (10 μg mL^-1^). The bacteria were pelleted by centrifugation at 1500 × g for 5 min, washed three times with sterile PBS, and resuspended in sterile PBS. The suspended bacterial pellet was sequentially incubated with anti-FLAG tag mouse monoclonal antibody (diluted 1:1,000) and FITC-conjugated anti-mouse IgG secondary antibodies (diluted 1:250). Fluorescence of the target protein expressed on the surface of *Lactobacillus* was measured by fluorescence microscopy.

### 2.7 Analysis of the MoDCs targeting ability of recombinant *Lactobacillus*

#### 2.7.1 Cultivation of recombinant *Lactobacillus*

Recombinant *Lactobacillus* were cultured overnight at 37°C in MRS broth at a ratio of 1:50. The bacterial pellet was collected by centrifugation at 1500 × g for 5 min, washed three times with sterile PBS, and resuspended in RPMI-1,640. The recombinant *Lactobacillus* was used to stimulate porcine MoDCs.

#### 2.7.2 Field-emission scanning electron microscope

MoDCs were cultured in 6-well plates with cell crawling for 6 days. The immature porcine MoDCs were incubated with 6aa-COE/*L. reuteri*, 87aa-COE/*L. reuteri*, COE/*L. reuteri*, and vector/*L. reuteri* were incubated in 5% CO_2_ at 37°C for 120 min. *Lactobacillus* that did not adhere were washed with sterile PBS. The samples were then processed using field-emission scanning electron microscopy.

#### 2.7.3 Flow cytometry

The four strains of cultured recombinant *Lactobacillus* were cultivated according to the above steps, resuspended in 500 µL PBS, loaded with an equal volume of carboxyfluorescein succinimidyl ester (CFSE; eBioscience, San Diego, CA, USA), and incubated for 30 min at 4°C. Bacteria were washed three times with sterile PBS. Then the labeled recombinant *Lactobacillus* were added to immature porcine MoDCs, incubated at 37°C for 30 min. After washing thrice with sterile PBS, the cells were subjected to flow cytometry. Immature porcine MoDCs were used as negative controls.

#### 2.7.4 Flat colony counting method

Four strains of cultured recombinant *Lactobacillus* were incubated with immature porcine MoDCs at 37°C for 30 min and washed three times with sterile PBS. Immature MoDCs were then collected in an Eppendorf tube for lysis. After centrifugation at 1500 × g for 5 min, the precipitate was collected and resuspended in 100 µL PBS. Thereafter, the precipitate was applied to the MRS broth plates with a spreading rod and cultured overnight at 37°C. After cultivation, the colonies were picked and incubated in MRS broth medium overnight at 37°C, PCR was used to identify the 6aa-COE gene in the recombinant plasmid of 6aa-COE/*L. reuteri*, 87aa gene in the recombinant plasmid 87aa-COE/*L. reuteri*, COE gene in the recombinant plasmid of COE/*L. reuteri*, and part of the vector gene in the recombinant plasmid of vector/*L. reuteri*. The colonies were counted and all measurements were performed in triplicates.

### 2.8 Quantitative real-time polymerase chain reaction (qRT-PCR) analysis of surface molecules, cytokines, and TLR mRNA expression in porcine MoDCs

The MoDCs were incubated with 6aa-COE/*L. reuteri*, 87aa-COE/*L. reuteri*, COE/*L. reuteri*, vector/*L. reuteri*, and lipopolysaccharide (LPS) at 37°C for 12 h. Total RNA was extracted from non-stimulated MoDCs and MoDCs incubated with LPS and recombinant *Lactobacillus* using an RNAprep pure Micro kit (TIANGEN, Beijing, China) according to the manufacturer’s instructions. The complementary DNA template for qRT-PCR was obtained by reverse transcription and amplification was performed as follows: 42°C for 2 h, 72°C for 10 min, and rested on ice for 5 min. Primers for the qRT-PCR analysis were designed using Primer 5 (**Table 2**). The SYBR Green PCR Master Mix (Roche, Shanghai, China) was used to perform qRT-PCR. Amplification and detection of specific products were performed using the 7500 Fast Real-Time PCR System (Applied Biosystems) with the following cycle profile: 95°C for 10 min, followed by 40 cycles of 95°C for 15 s and 60°C for 1 min. β-actin was used as an internal control. Relative mRNA expression was calculated using the formula delta Ct (ΔΔCt) = [Ct _(stimulated-MoDCs, target_ gene)-Ct _(stimulated-MoDCs, β-actin)_] - [Ct _(non-stimulated-MoDCs, target_ gene)-Ct _(non-stimulated-MoDCs, β-actin)_]. The results were calculated by 2^-ΔΔCt^.

**Table 2:** Primers used in quantitative real-time PCR.

Primers	Sequence (5′-3′)	GenBank ID
β-action	F: GGACTTCGAGCAGGAGA R: AGGAAGGAGGGCTGGAAGAG	NM_205518.1
CD40	F: CGTGCGGGGACTAACAAGA R: CCAACAGGACGGCAAACA	NM_214194.1
CD80	F: GAGTCCGAATATACTGGCAAAAGG R: AGGTGCGGTTCTCATACTTGG	KP342302.1
CD86	F: GTGTGGGATGGTGTCCTTTGT R: TTTGTTCACTCGCCTTCCTGT	KF646138.1
TLR-2	F: ACCATTCCCCAGCGTTTCT R: GAGTCAGCAAGTCACCCTTTATGTT	NM_213761.1
TLR-4	F: ACCAGACTTTCTTGCAGTGGGTCA R: AATGACGGCCTCCrCTTATCTGACA	KF460453.1
TLR-6	F: TCCCAGAATAGGATGCAGTGCCTT R: ACTCCTTACATATGGGCAGGGCTT	NM_213760.2
TLR-9	F: ACCAGGGACAACCACCACTT R: CAGGCAGAGAGGCAAATCC	XM_005669564.3
IL-12	F: TCAGAAGGCCAAACAAACCCT R: GGCAACTCTCATTCGTGGCTA	NM_213993.1
IL-10	F: TCTGAGAACAGCTGCATCCAC R: CGCCCATCTGGTCCTTCGTT	NM_214041.1
IFN-γ	F: CGCAAAGCCATCAGTGAACTCA R: TCTGGCCTTGGAACATAGTCT	HQ026021.1

### 2.9 Evaluation of mixed leukocyte reaction (MLR) in MoDCs

MLR was evaluated using an automated ELISA reader (Bio-Tech Instruments, USA) and CCK-8 cell counting kit (Beyotime, China). Preparation of reaction cells: PBMCs were obtained from piglet peripheral blood by density gradient centrifugation, resuspended in RPMI-1640 basal culture medium, and washed thrice. The cell concentration was adjusted to approximately 2 × 10^6^ mL^-1^ using RPMI-1640 complete culture medium. For preparation of stimulating cells immature MoDCs were incubated with LPS or 6aa-COE/*L. reuteri*, 87aa-COE/*L. reuteri*, COE/*L. reuteri*, or vector/*L. reuteri* and treated with 25 µg/mL final mass concentration mitomycin C, incubated at 37°C for 1 h, resuspended in RPMI-1640 basal culture medium, and washed three times. In a 96-well plate, T-lymphocytes were co-cultured with recombinant *Lactobacillus*-incubated DCs (1 × 10^5^ cells/well) at ratios of 1:1, 1:10, or 1:100 (DCs: lymphocytes). Negative control wells were used for MoDCs and T-cells, and blank control wells were used for RPMI 1640 culture solution. Three replicates per well were incubated at 37°C in a 5% CO_2_ incubator for 72 h. Finally, CCK-8 was added to the 96-well plates, and OD450 values were read on an ELISA reader. The stimulation index (SI) was calculated using the following formula: SI = (OD_sample_-OD_stimulator cells only_)/(OD_responder cells only_-OD_blank control_).

### 2.10 Detection of cytokines in Mo-DCs by ELISA

To detect the secretion of cytokines, supernatants were obtained from immature or mature MoDCs (5× 10^5^ in 6-well plates) that had been treated with recombinant *Lactobacillus* at a concentration of 10 TCID50 cell^−1^ or LPS (2 μg mL^−1^) for 24 h. Levels of secreted IL-10, IL-12, and IFN-γ (R&D Systems, USA) were determined using commercial ELISA kits according to the manufacturer’s recommendations and calculated based on the curve generated using the cytokine standards.

### 2.11 Structural analysis of the protein

The crystal structure of porcine CTLA4 needed to be determined. The 3D structural model of porcine CTLA4 protein was generated from the crystal structure of human CTLA4/B7 protein complex (PDB code: 1I8L) template using Phyre2 (https://www.sbg.bio.ic.ac.uk/phyre2). Sequence alignment of porcine CTLA4 and human CTLA4 was performed using NCBI BLAST (https://blast.ncbi.nlm.nih.gov/Blast.cgi). The binding interface between porcine CTLA4-6aa, CTLA4-87aa, and B7 molecules was analyzed based on the human CTLA4/B7 complex protein model using the PyMOL software (31).

### 2.12 Immunization and specimen collection

One-month-old specific pathogen-free (SPF) piglets were housed under SPF conditions with free access to a standard chow diet and water. Before oral administration, the recombinant *Lactobacillus* was cultured overnight in MRS medium, washed with PBS, and resuspended in PBS at a final concentration of 1 × 10^10^ CFU/mL. The piglets were randomly divided into three groups, with three piglets per group.

The first group was orally administered with 5 mL of recombinant *Lactobacillus* COE/*L. Reuteri* (1 × 10^10^ CFU/mL). The second group was orally administered with 5 mL of recombinant *Lactobacillus* 6aa-COE/*L. Reuteri* (1 × 10^10^ CFU/mL). The third group, which served as an unimmunized control, was inoculated with 5 mL of orally administered PBS. The piglets were immunized orally once a day for three consecutive days on days 1, 2, and 3. Serum, nasal, and anal swab samples were obtained on days 0, 7, 14, 21, 28, 35, 42, and 47 after immunization and were stored at −20°C until use.

### 2.13 ELISA immunoassay

The levels of anti-COE-specific IgG in the sera and SIgA in the sera, nasal cavity, and feces of the piglets used in this study were determined using ELISA. Briefly, polystyrene microtiter plates were coated with PEDV and incubated overnight at 4°C. After washing thrice with PBST (PBS containing 1% Tween-20), the plates were blocked with 5% skim milk at 37°C for 2 h. After washing thrice with PBST, the collected samples, prepared in triplicate, were diluted with PBS and incubated at 37°C for 1 h. Then, a conjugated goat anti-pig IgG or IgA antibody (Abcam, Cambridge, UK) was added to each well (1:5000) and incubated for 1 h at 37°C. The color was developed using tetramethylbenzidine (TMB; Sigma, Ronkonkoma, NY, USA) as a substrate, and absorbance was measured at 490 nm.

### 2.14 Statistical analysis

The experimental data were statistically analyzed using GraphPad Prism (version 8.0.2) statistical software. Data were presented as mean ± standard deviation (SD) and analyzed using two-way ANOVA with multiple comparison (LSD) tests using SPSS. Different letters (a vs. b, a vs. c, and b vs. c) indicate significant differences (*p* < 0.01) at the same time point.

## 3 Results

### 3.1 Cells generated by culturing porcine PBMCs with recombinant porcine GM-CSF and IL-4 have the morphology and phenotype of typical DCs

Porcine PBMCs were cultured in the presence of recombinant porcine granulocyte-macrophage colony-stimulating factor (GM-CSF) and IL-4. Changes in cell morphology were observed on days 1, 2, 4, and 6 using an optical microscope. As shown in **Figure S2A**, the isolated monocytes were round in shape and cell aggregates gradually appeared on day 2. As these cell aggregates grew, the surfaces of some cells displayed a veiled and dendritic appearance on day 4, and most cells displayed a typical immature DC appearance on day 6. Fluorescence microscopy and flow cytometry were used to analyze the phenotypes of immature porcine MoDCs. A total of 94.8% of cells stained positive for anti-porcine MHC class II, and 77.1% of cells stained positive for anti-porcine CD172a (**Figure S2B**). Immature porcine MoDCs expressed high levels of MHC class II molecules and CD172a (**Figure S2C**). This suggests that GM-CSF and IL-4 treatment facilitated the differentiation of PBMCs into immature porcine MoDCs.

### 3.2 CTLA4-6aa binds to porcine DCs

**Figure 1:**
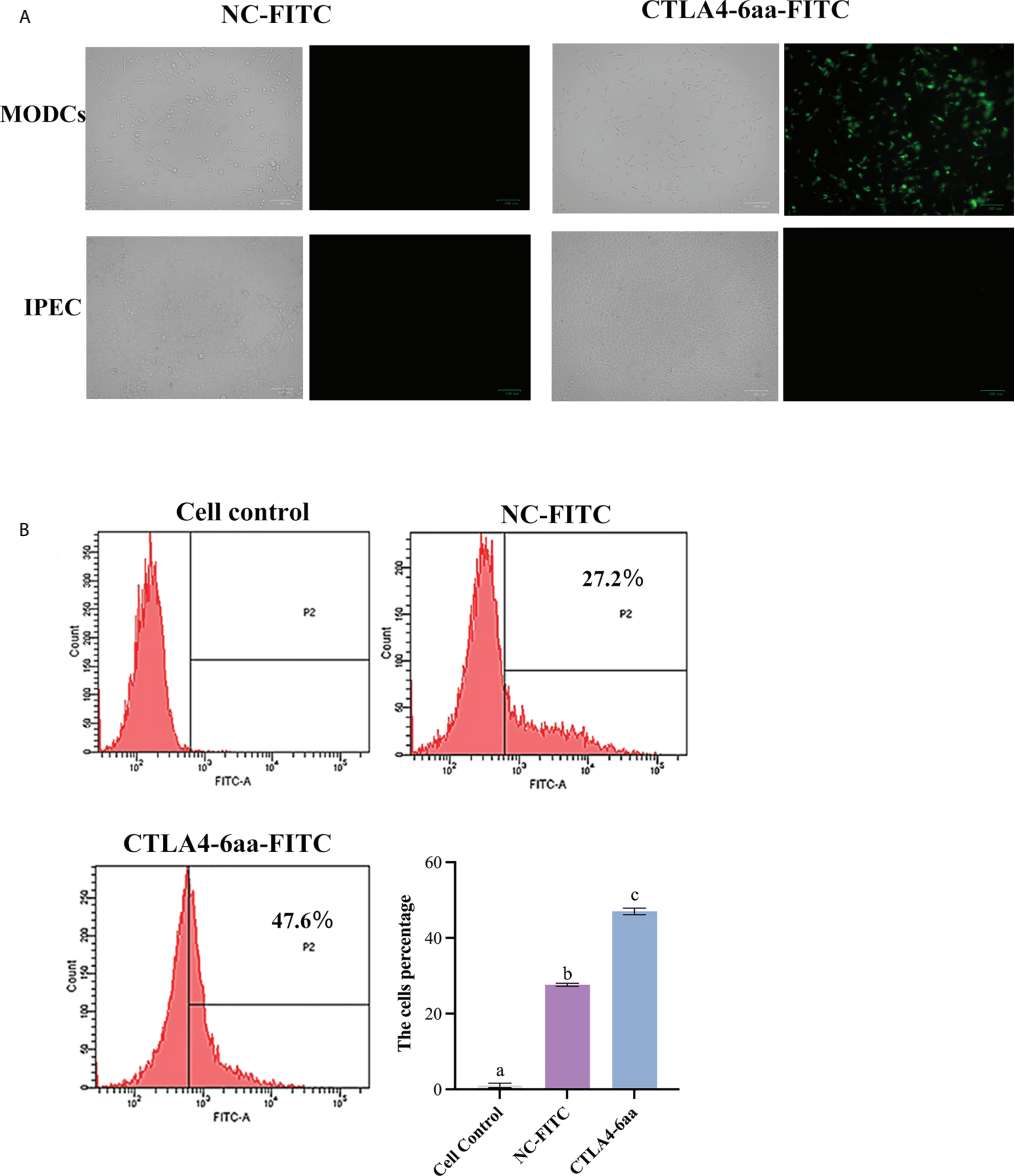
Ability of CTLA4-6aa-FITC to bind porcine monocyte derived dendritic cells (MoDCs) was analyzed by fluorescence microscopy and flow cytometry. Fluorescence microscopy: CTLA4-6aa-FITC is shown in green. Scale bar = 100 µm **(A)**. Flow cytometry: CTLA4-6aa-FITC counts and binding of NC-FITC (as negative control) to porcine MoDCs, and the mean fluorescence intensities of FITC-labeled peptides binding to DCs **(B)**. Different letters (a vs. b, a vs. c, b vs. c) indicate significant differences (*p* < 0.01) at the same time point.

The ability of CTLA4-6aa to bind to porcine MoDCs was determined using fluorescence microscopy. We observed that the green fluorescence of porcine MoDCs incubated with FITC-modified CTLA4-6aa was higher than that of MoDCs incubated with the FITC-modified negative control (NC) and no green fluorescence from IPECs incubated with FITC-modified CTLA4-6aa was observed (**Figure 1A**). For accurate quantitative analysis, flow cytometry was used to evaluate the aforementioned binding ability. The results showed that the positive rate of porcine MoDCs incubated with CTLA4-6aa-FITC was 47.6%, which was significantly higher than that of MoDCs from the NC-FITC and control groups (**Figure 1B**). These results indicate that CTLA4-6aa can bind porcine DCs.

### 3.3 Recombinant *Lactobacillus* expressing partial porcine CTLA4 motifs and PEDV protective antigen

**Figure 2:**
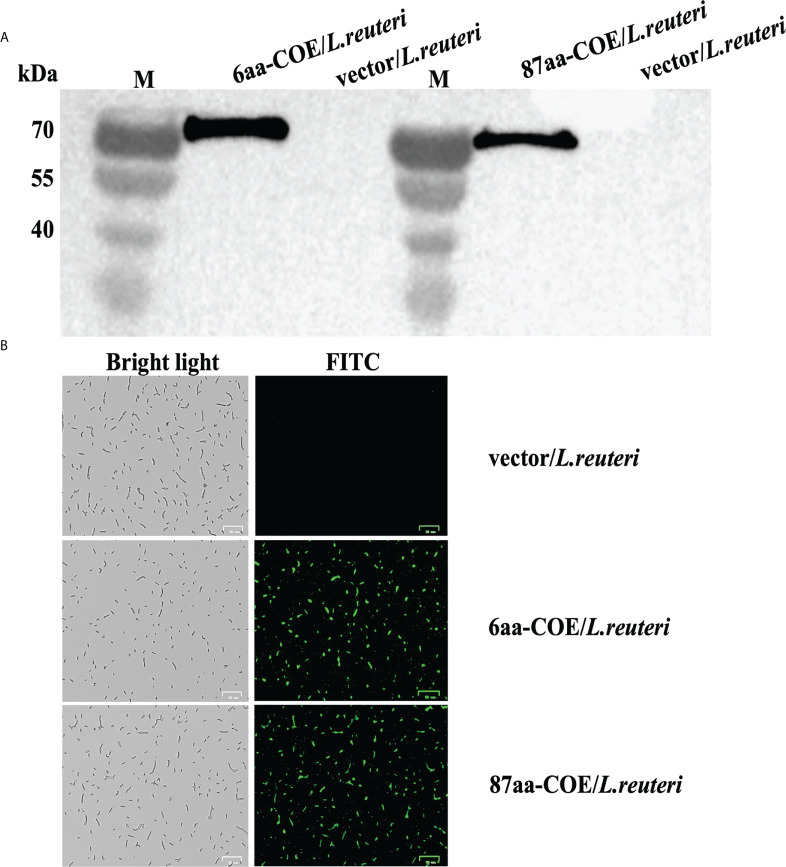
Western blotting analysis for identifying the expression of recombinant proteins 6aa-COE and 87aa-COE in *Lactobacillus* (Mouse anti-Flag monoclonal antibody was used as the primary antibody) **(A)**. Identification of recombinant proteins expressed in *Lactobacillus* through fluorescence microscopy **(B)**.

To evaluate the ability of partial porcine CTLA4 motifs and antigens to bind DCs, recombinant *Lactobacillus* expressing CTLA4-6aa or CTLA4-87aa with PEDV protective antigen were constructed in this study. Expression of the recombinant proteins, namely, 6aa-COE and 87aa-COE, were confirmed by western blotting. As shown in **Figure 2A**, the fusion proteins 6aa-COE (left, 66 kDa) and 87aa-COE (right, 71 kDa) were detected in the immunoblots. The protein size was consistent with the predicted molecular weight (the protein size of the polyglutamate synthase A (PgsA) anchor was approximately 45 kDa, 6aa-COE was approximately 21 kDa, 87aa-COE was approximately 26 kDa). The NC vector/*L. reuteri* did not produce the corresponding immunoreactive bands. An indirect immunofluorescence assay was used to determine protein expression in recombinant *Lactobacillus*. **Figure 2B** shows that green fluorescence was detected in 6aa-COE/*L. reuteri* and 87aa-COE/*L. reuteri* compared to vector/*L. reuteri*. Therefore, the recombinant proteins were expressed in the *Lactobacillus*.

### 3.4 Recombinant *Lactobacillus* expressing CTLA4-6aa recognized and captured by porcine MoDCs with increased efficiency

**Figure 3:**
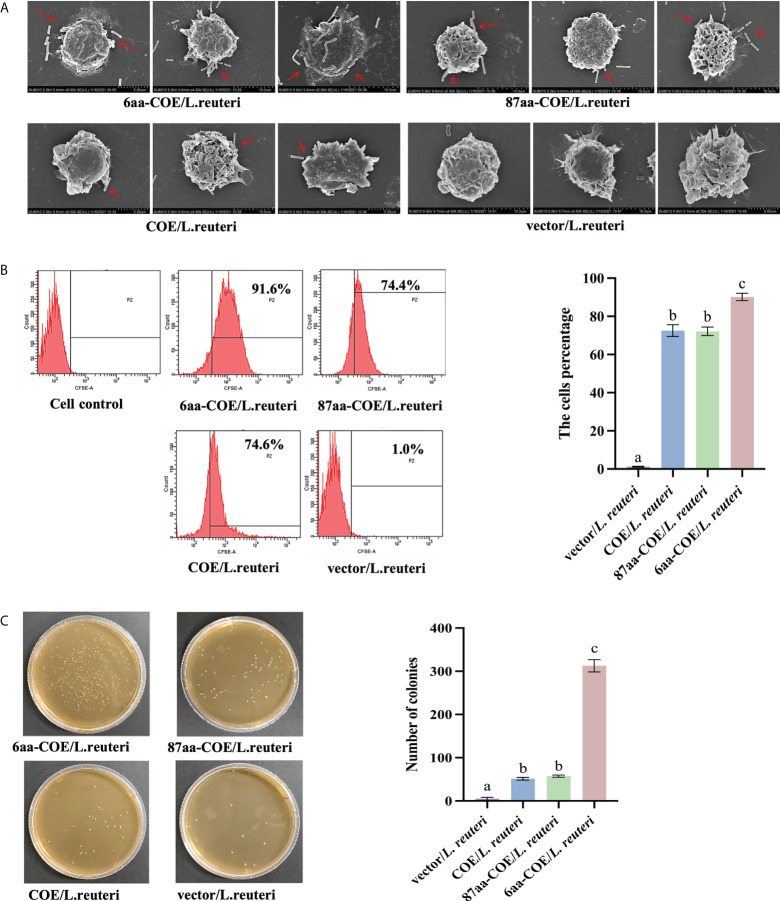
Scanning electron micrographs demonstrate the recombinant *Lactobacilli* recognized and captured by porcine monocyte derived dendritic cells (MoDCs) **(A)**. Flow cytometry results of recombinant *Lactobacillus* recognized and captured by porcine MoDCs, and the mean fluorescence intensities of recombinant *Lactobacillus* recognized and captured by porcine MoDCs **(B)**. Plate count assay for measuring the ability of recombinant *Lactobacillus* to target porcine MoDCs **(C)**. Different letters (a vs. b, a vs. c, b vs. c) indicate significant differences (*p* < 0.01) at the same time point.

Field-emission scanning electron microscopy was used to evaluate the efficiency with which recombinant *Lactobacillus* were captured and recognized by porcine MoDCs. The results showed that while 6aa-COE/*L. reuteri*, 87aa/*L. reuteri*, and COE/*L. reuteri* were recognized and captured by porcine MoDCs, vector/*L. reuteri* could not be captured by porcine MoDCs. Moreover, 6aa-COE/*L. reuteri* was more efficiently recognized and captured by porcine MoDCs than 87aa-COE/*L. reuteri* and COE/*L. reuteri*. There was no difference between the 87aa-COE/*L. reuteri* and COE/*L. reuteri* groups with regards to the efficiency of capture and recognition (**Figure 3A**).

For a more accurate quantitative analysis, flow cytometry and flat colony counting were used to assess the efficiency with which recombinant *Lactobacillus* was captured and recognized by porcine MoDCs. Flow cytometry showed that counts of 6aa-COE/*L. reuteri* (captured by porcine MoDCs) were higher than that of the 87aa-COE/*L. reuteri* and COE/*L. reuteri* groups (*p*<0.01). The counts of 87aa-COE/*L. reuteri* and COE/*L. reuteri* groups (*p*>0.05) did not differ significantly (**Figure 3B**). The results of the flat colony counting were consistent with those obtained by flow cytometry (**Figure 3C**). Together, these results indicated that recombinant *Lactobacillus* expressing CTLA4-6aa was recognized and captured by porcine MoDCs with increased efficiency. Therefore, CTLA4-6aa, not CTLA4-87aa, expression was able to enhance the capture and recognition of recombinant *Lactobacillus* by porcine MoDCs.

### 3.5 Recombinant *Lactobacillus* expressing CTLA4-6aa up-regulate the mRNA expression of markers, cytokines, and TLRs in DCs

**Figure 4:**
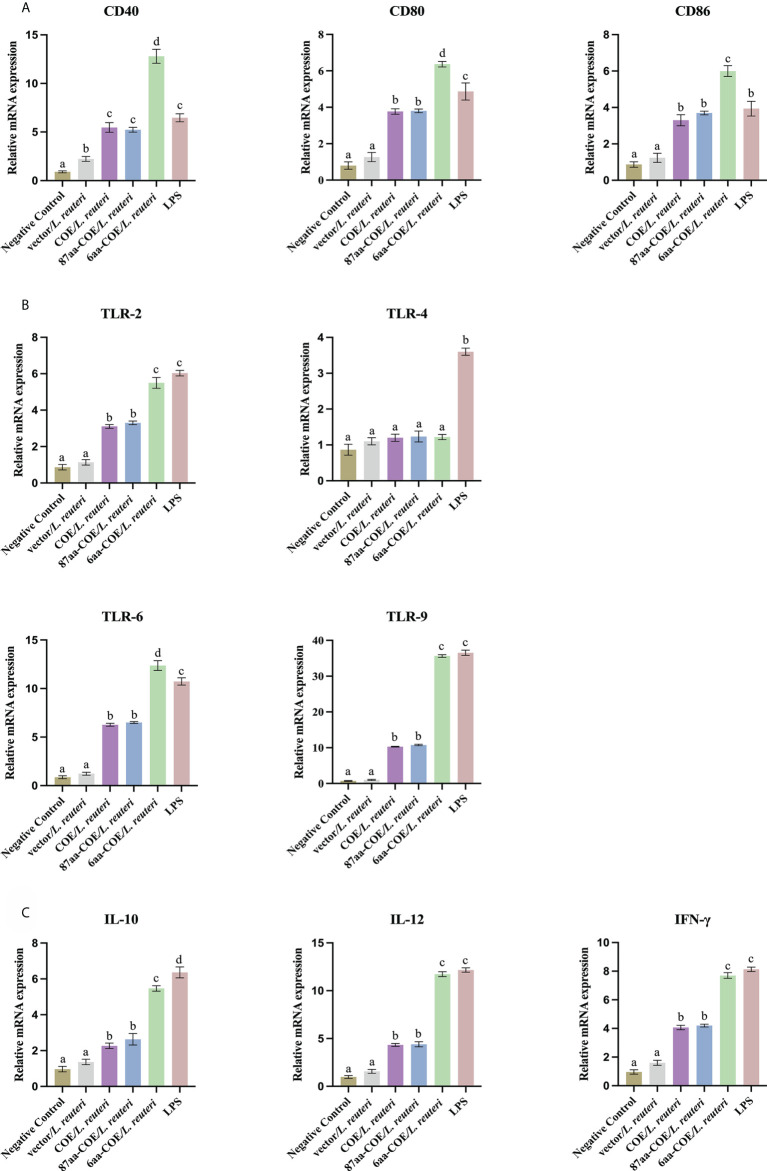
Expression of surface molecules, cytokines, and toll-like receptors (TLRs) by porcine monocyte derived dendritic cells (MoDCs) was assessed by relative quantitative real-time polymerase chain reaction (qRT-PCR). The expression of surface molecules on porcine MoDCs after stimulation with four recombinant *Lactobacillus* and lipopolysaccharide (LPS) was assessed by relative qRT-PCR **(A)**. Expression of cytokines by porcine MoDCs stimulated with four recombinant *Lactobacillus* and LPS was assessed by relative qRT-PCR **(B)**. Toll-like receptors expression by porcine MoDCs stimulated by four recombinant *Lactobacillus* and LPS was assessed by relative qRT-PCR **(C)**. Different letters (a vs. b, a vs. c, b vs. c) indicate significant differences (*p* < 0.01) at the same time point.

DC maturation is critical for priming the immune response. It is characterized by an increase in the expression of DC markers and TLRs, and increased secretion of cytokines. The results of qRT-PCR showed that the levels of DCs markers (CD40, CD80, and CD86), TLRs (TLR-2, 6, and 9), and cytokines (IL-10, IL-12, and IFN-γ) were significantly higher in 6aa-COE/*L. reuteri*-stimulated porcine DCs compared to DCs treated with 87aa-COE/*L. reuteri* (*p*<0.01). Expression of these markers did not differ markedly between the 87aa-COE/L. reuteri and COE/L. reuteri groups (**Figures 4A–C**). These results suggest that recombinant *Lactobacillus* expressing CTLA4-6aa may promote DC maturation.

### 3.6 Fusion of CTLA4-6aa with antigens promotes DC-mediated T-cell differentiation into Th1 cells

**Figure 5:**
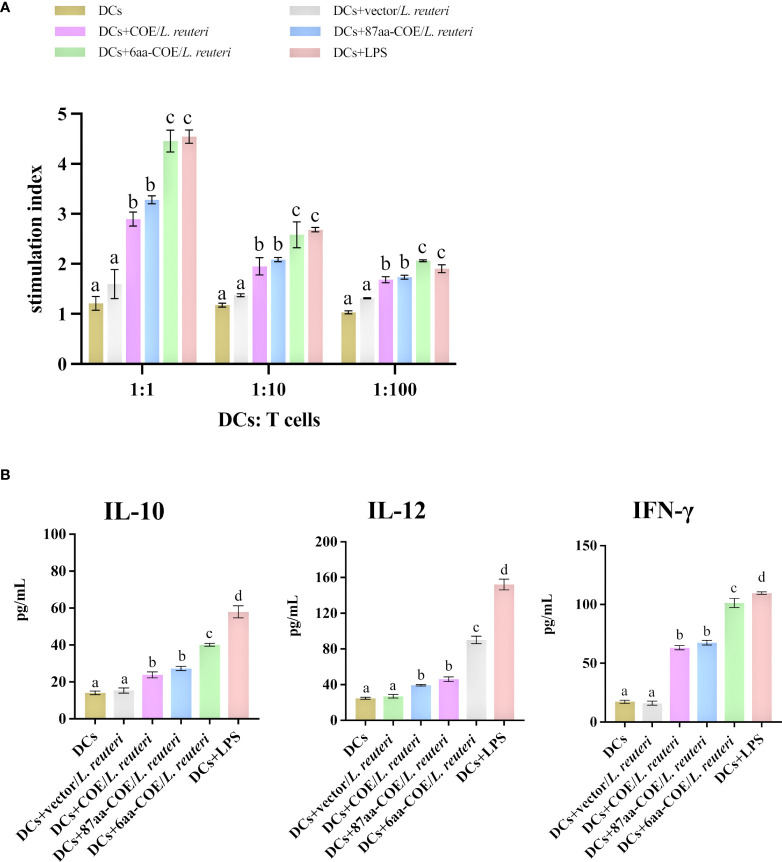
Analysis of MoDCs treated with four recombinant *Lactobacillus* and LPS skew T-cells toward different effector T-cell profiles. **(A)** MoDCs treated with recombinant *Lactobacillus* and LPS stimulate the proliferation of T-lymphocytes in mixed lymphocyte reaction (MLR). Responder cells were added in ratios of 1:1, 1:10, or 1:100 and co-cultured with the stimulated cells for 72 h. Proliferation is expressed as the Stimulation Index (SI) calculated with the formula: SI = (OD_sample_ − OD_stimulator cells only_)/(OD_responder cells only_ − OD_blank control_). All experiments were performed in triplicate at a minimum. Data are presented as mean ± SEM (n = 6 per group) **(A)**. MoDCs were stimulated with recombinant *Lactobacillus* and LPS for 12 h and then co-cultured with allogenic T-cells at a ratio of 1:1. After 72 h, culture supernatants were collected and analyzed for cytokines by ELISA **(B)**. Different letters (a vs. b, a vs. c, b vs. c) indicate significant differences (*p* < 0.01) at the same time point.

To assess whether the fusion of CTLA4-6aa with antigens affects antigen presentation ability, we prepared single-cell suspensions of lymphocytes from piglets and performed cell proliferation tests. The results showed that 6aa-COE/*L. reuteri* resulted in significantly higher levels of T-cell proliferative responses than COE/*L. reuteri* (*p*<0.01) (**Figure 5A**). This result implies that the fusion of CTLA4-6aa with antigens enhanced the ability of porcine DCs to activate T-cell proliferation.

To further determine the type of T-cell differentiation that is mediated by MoDCs stimulated with recombinant *Lactobacillus*, we measured the levels of IFN-γ, IL-12, and IL-10 using ELISA kits. **Figure 5B** shows that 6aa-COE/*L. reuteri* induced the secretion of a significant number of cytokines (IFN-γ, IL-12, and IL-10) by DCs and T-cells (*p*<0.01). In addition, the secretion of IFN-γ and IL-12 was higher than that of IL-10 by MoDCs induced by recombinant *Lactobacillus*. From the above results, it can be concluded that, with the participation of CTLA4-6aa, recombinant *Lactobacillus* stimulates MoDCs to mediate the differentiation of more T-cells into Th1 cells.

### 3.7 Forming intramolecular hydrogen bonds between CTLA4-87aa residues reduce binding to the B7 molecule on DCs

**Figure 6:**
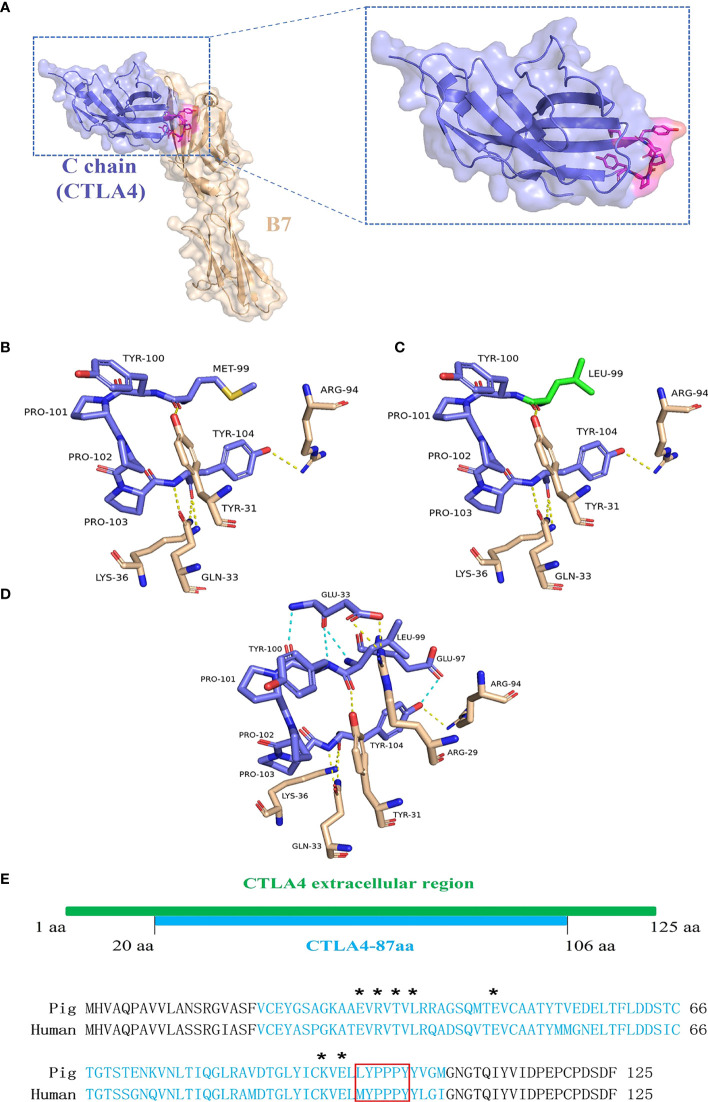
Analysis of porcine CTLA4 protein structure and binding interface. The porcine protein structure was generated from the crystal structure of the human CTLA4/B7 complex’s C chain (Protein Data Bank accession code 1I8L) templated by the Phyre2. **(A)** Ribbon diagram of the human CTLA4/B7 complex showing CTLA4 (purple), B7 (wheat), and MYPPPY motif (magenta). **(B)** Structure of the human MYPPPY motif bound to B7 (shown in sticks, colored by atom type). The yellow dotted line indicates hydrogen bonds between the residues. **(C)** Structure of porcine CTLA4-6aa (LYPPPY) bound to B7 (shown in sticks, colored by atom type). The green sticks represent the residue Leu 99, which was generated by mutating Met 99 and using the PyMOL software. **(D)** Structure of porcine CTLA4-87aa bound to B7 (shown in sticks, colored by atom type). The intramolecular hydrogen bonds (cyan dotted line) are shown. **(E)** Sequence alignment of porcine and human CTLA4 extracellular regions. Porcine CTLA4-87aa sequences are labeled in cyan. Asterisks denote the Glu 33, Arg 35, Thr 37, Leu 39, Glu 48, Lys 95, and Glu 97 residues. The LYPPPY and MYPPPY motifs are boxed in red.

The porcine CTLA4 protein structure was simulated using Phyre2 based on the crystal structure of the human CTLA4/B7 complex protein model. As shown in **Figure 6A**, structural homology searches using Phyre2 suggested that the C chain of the human CTLA4/B7 complex is most similar to the porcine CTLA4 extracellular region, with MYPPPY serving as the CTLA4/B7 binding interface. In addition, the porcine CTLA4 sequence showed 87.6% homology to the human CTLA4 sequence.

The CTLA4/B7 binding interface was analyzed using PyMOL software and was based on the human CTLA4/B7 complex protein model. As shown in **Figure 6B**, the binding interface of the human MYPPPY motif interacted with B7 *via* five hydrogen bonds between the Met 99 and Tyr 104 residues on human CTLA4. Since the Met at position 99 was replaced by Leu in the case of the porcine CTLA4 sequence, residue conversion was performed using PyMOL software, and Leu substitutions were selected for 3D structure analysis. The connections between the replaced residues and changes in hydrogen bonds are depicted in **Figures 6C** and **Table 3**. The hydrogen bond between Leu 99 on porcine CTLA4 and Tyr 31 on B7 remained unchanged. For the porcine CTLA4-87aa protein, some residues (Glu 33, Arg 35, Thr 37, Leu 39, Glu 48, Lys 95, and Glu 97) speculated by Metzler et al. (1997) may show the importance of DCs binding, which is conserved in both human and porcine sequences (**Figure 6E**). Notably, as shown in **Figure 6D** and **Table 4**, intramolecular hydrogen bonds were formed between Glu 33 and Leu 99, Glu 33 and Tyr 100, and Glu 97 and Tyr 104 residues in the CTLA4-87aa protein, whereas intermolecular hydrogen bonds were formed between Glu 33 and Arg 29 residues. These results indicate that the intramolecular hydrogen bonds in CTLA4-87aa may weaken the intermolecular forces between porcine CTLA4 and B7 residues, thereby negatively affecting the binding of CTLA4 and B7.

**Table 3:** Analysis of the changes in hydrogen bonds following non-synonymous mutations in the Leu 99 residue of (3D protein structure) porcine CTLA4.

Residues (Human CTLA4)	Number of hydrogen bonds	Binding residues (B7)		Residues (Porcine CTLA4)	Number of hydrogen bonds	Binding residues (B7)
Met 99	1	Tyr 31		Leu 99	1	Tyr 31
Tyr 104	3	Gln 33 Lys 36 Arg 94		Tyr 104	3	Gln 33 Lys 36 Arg 94

**Table 4:** Analysis of the hydrogen bonds between porcine CTLA4-87aa and B7.

Binding Residues (B7)	Number of Intermolecular hydrogen bonds	Residues (CTLA4)	Number of intramolecular hydrogen bonds	Binding residues (CTLA4)
Arg 29	2	Glu 33	3	Leu 99 Tyr 100
		Glu 97	1	Tyr 104

### 3.8 IgG and SIgA levels induced by recombinant *Lactobacillus reuteri via* oral immunization

**Figure 7:**
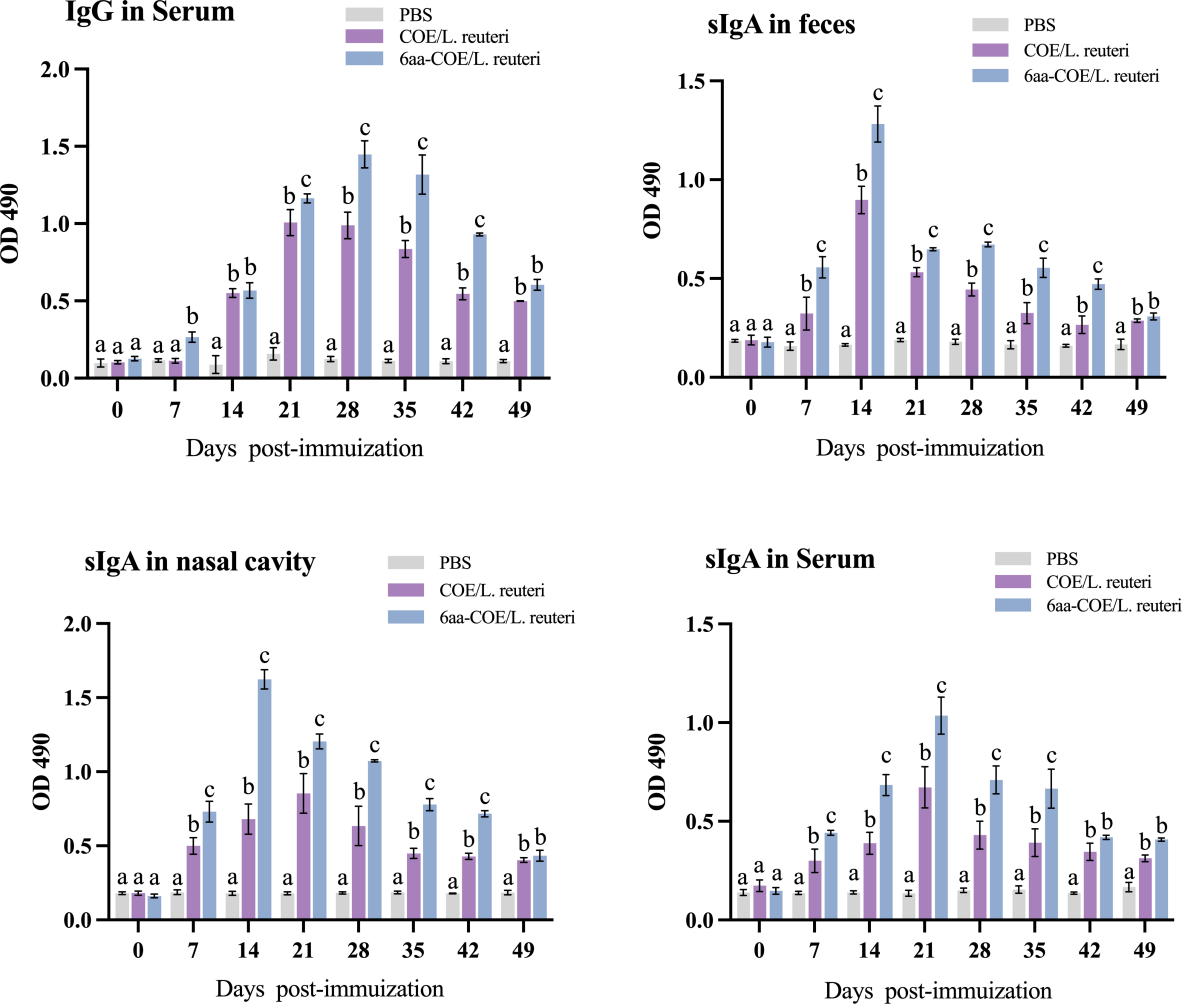
Specific anti-PEDV IgG and sIgA antibody levels in piglets orally immunized with the PBS, recombinant *Lactobacillus* 6aa-COE/*L. reuteri* and COE/*L. reuteri*. Measurement of a specific anti-PEDV IgG antibody in the anti-sera from immunized piglets by ELISA using PEDV as the coating antigen. Measurement of specific anti-PEDV SIgA antibody levels in the feces, nasal cavity, and serum by ELISA using PEDV as the coating antigen. Different letters (a vs. b, a vs. c, b vs. c) indicate significant differences (*p* < 0.01) at the same time point.

Anti-PEDV-specific IgG was assessed to evaluate the ability of recombinant *Lactobacillus* strains to induce humoral immune responses in piglets. Serum was collected from piglets between days 0 and 49 post-immunization (collected every seven days), and serum-specific IgG responses were measured using ELISA. As shown in **Figure 7**, piglets in the 6aa-COE/*L. reuteri* group showed the highest specific IgG levels. From the fourteenth day after vaccination, a significant level of anti-PEDV-specific IgG antibody was induced in piglets that were orally administered 6aa-COE/*L. reuteri* (*p*<0.01) than COE/*L. reuteri* groups. The level of anti-PEDV-specific IgG antibody was the highest 28 days post-vaccination, significantly higher than in the other groups. This result shows that 6aa-COE/*L. reuteri* can elicit specific systemic immunity.

The intestinal mucosa cell-mediated immune response was further evaluated by measuring the specific anti-PEDV SIgA antibody in the nasal cavity, feces, and sera collected from immunized piglets post-immunization using ELISA. Mucosal SIgA levels increased after oral immunization with COE/*L. reuteri* and 6aa-COE/*L. reuteri* in the nasal cavity, feces, and sera (*p*<0.01) than those in the control groups of piglets orally immunized with PBS (**Figure 7**). Anti-PEDV SIgA in the feces was detected as early as the seventh-day post-immunization with COE/*L. reuteri* and 6aa-COE/*L. reuteri*. In addition, the level of SIgA antibody was induced by 6aa-COE/*L. reuteri via* oral immunization which was significantly higher than that induced by the COE/*L. reuteri*.

## 4 Discussion

The molecules on the surface of DCs recognize antigens and enable antigen uptake to facilitate the induction of strong immune responses (32). Therefore, the efficiency of antigen capture by DCs can be improved by the expression of molecules that directly target these cells. CTLA4 can specifically bind B7 molecules on the surface of DCs. Furthermore, the extracellular region of CTLA4 has been identified as the main domain that binds B7 (15). A few key motifs may be crucial for mediating the interaction between the receptor and its ligands. A previous study hypothesized that a peptide motif (MYPPPY) in the extracellular region of human CTLA4 may play an important role in binding the B7 molecule on DCs, but no formal studies have been performed to verify the above speculations (20). Based on these aforementioned study, we identified the key motifs in porcine CTLA4 that are important for binding the B7 molecule to porcine DCs. Thereafter, *Lactobacillus* expressing these key motifs in combination with the antigen were used to analyze the efficiency of antigen capture and recognition by DCs and monitor their effect on DC function. Our study may promote the targeting of DCs as a potential strategy for the development of porcine vaccines.

For this DC-targeting strategy mentioned above, the expansion of DCs for *in vitro* studies is necessary because they are rare in body tissues, and in mammals many DCs are generated from blood or bone marrow-derived DCs (33, 34). At present, the isolation of monocytes from peripheral blood is a simple and convenient procedure. Consequently, we generated and used MoDCs in our study. Specifically, PBMCs were cultured in the presence of recombinant porcine GM-CSF and IL-4 for 6 days, and the cultured cells had the morphology of typical DCs. A majority of the cultured cells express MHC class II and CD172a, which are necessary signal transduction molecules for the recognition and capture of antigens by DCs (35, 36).

DC-targeting strategies have become the focus of research, as DCs play a significant role in the induction and regulation of immune responses. The strategy described in our study can be used to develop DC-targeting vaccines to increase antigen availability for DCs (37). To increase the bioavailability of immunogens in pigs, a porcine DC-targeting peptide was identified in this study. Our study indicated that the LYPPPY motif can bind porcine MoDCs and may serve as a suitable alternative for developing targeted vaccines in porcine. In addition, to evaluate the ability of the LYPPPY motif in the extracellular domain of porcine CTLA4 to target DCs, we analyzed the amino acids flanking the LYPPPY motif. Thereafter, recombinant *Lactobacillus* vaccines with CTLA4-6aa and CTLA4-87aa were constructed to evaluate whether the targeted sequences could direct antigens to DCs.

Choosing suitable antigen delivery vectors is key to evaluating the efficiency of DCs targeting. *Lactobacillus* in the intestinal tract that can express and deliver antigens (38). They are good vectors substances for studying antigens directly on DCs (39). In particular, *Lactobacillus* have probiotic properties and can resist gastric acid bile salts and colonize the intestine (40). In this study, a *Lactobacillus* expression vector (pPG-T7g10-PPT) was used to express DC-targeting sequences and antigens. This vector contains the polyglutamate synthase A (PgsA) anchor protein, which is a transmembrane protein that can be fused with the N-terminus of target proteins such that the target proteins are expressed on the bacterial surface (41). To evaluate the effectiveness of the DC-targeting sequences, recombinant *Lactobacillus*, namely 6aa-COE/*L. reuteri* and 87aa-COE/*L. reuteri* were constructed. This study showed that while recombinant *Lactobacillus* expressing CTLA4-6aa could efficiently target antigens to DCs, those expressing CTLA4-87aa had no obvious targeting effect compared to the recombinant *Lactobacillus* expressing COE only. Additionally, we analyzed the protein structure in our study. Analysis of the human CTLA4/B7 complex suggested that the CTLA4 and B7 ligand-binding sites were distal to the binding interface, providing the potential for B7 to bind CTLA4 (42). Notably, it is known that the ^99^MYPPPY^104^ motif in human CTLA4 is essential for its interaction with B7. In case of porcine CTLA4, Met 99 in the ^99^MYPPPY^104^ motif was replaced by Leu 99. The 3D structural simulation revealed that residue substitutions did not alter the interaction and stability of the CTLA4 protein structure. Therefore, it is plausible that the structure of the human CTLA4/B7 complex could serve as a reasonable model for studying the binding interface of porcine CTLA4. Our analysis using PyMOL software suggested that porcine CTLA4 binds to B7 *via* the ^99^LYPPPY^104^ motif, with Leu 99 and Tyr 104 being the key residues that bind B7. Notably, the unusual *cis-trans-cis* backbone conformation of the ^101^Pro-Pro-Pro^103^ sequence in the ^99^LYPPPY^104^ motif of CTLA4 contributes to the considerable geometric complementarity between CTLA4 and B7 (43). Together with the other experimental results in this study, these results show that CTLA4-6aa is a crucial site for binding B7. The binding interface between CTLA4-87aa and B7 was analyzed using a protein structure model. In case of the CTLA4-87aa sequence, the conserved residues (Glu 33, Arg 35, Thr 37, Leu 39, Glu 48, Lys 95, and Glu 97) and two hydrogen bonds between the Glu 33 residue on CTLA4 and the Arg 29 residue on B7 are likely to contribute to the specificity of binding (44). However, intramolecular hydrogen bonds were formed in the CTLA4-87aa protein between the Glu 33 and Leu 99, Glu 33 and Tyr 100, and Glu 97 and Tyr 104 residues. The formation of intramolecular hydrogen bonds may weaken the intermolecular forces between the residues on porcine CTLA4 and those on B7. This negatively affects the interaction between CTLA4 and B7.

Antigens are captured and processed by DCs before being presented to immune cells. DCs gradually mature, with increased expression of surface molecules and TLRs, and augmented cytokine secretion are characteristics of matured DCs (45). TLRs on the surface of DCs can also mediate their maturation and play an important role in initiating innate immune responses (46). Moreover, DCs secrete cytokines that perform different functions and facilitate immune response regulation (47). They have a critical effect on the differentiation of naïve T-cells into T-helper type 1 and type 2 cells (48, 49). Although IL-12 and IFN-γ are pro-inflammatory cytokines, IL-10 is an anti-inflammatory cytokine. The balance between both, the pro- and anti-inflammatory cytokines can regulate the immune response and exert anti-infection and anti-tumor functions (50). The results showed that the expressions of DCs surface molecules (CD40, CD80, and CD86), TLRs (TLR-2, 6, and 9), and cytokines (IL-10, IL-12, and IFN-γ) in 6aa-COE/*L. reuteri*-stimulated porcine DCs were significantly higher than that in DCs treated with 87aa-COE/*L. reuteri*. Notably, recombinant *Lactobacilli* had no effect on TLR-4 expression because TLR-4 can mainly recognize lipopolysaccharides from gram-negative bacteria and its expression may not be affected by gram-positive bacteria (51). A previous study reported DC-targeted peptide-conjugated antigen immunization to preferentially induce the expansion and proliferation of CD4^+^ T-cells (52). Our results align with previous studies in which recombinant *Lactobacillus* 6aa-COE/*L. reuteri* significantly induced MoDCs to stimulate T-cell proliferation at a DC/T ratio of 1:1. The balance between the Th1 and Th2 responses was evaluated based on the secretion levels of these cytokines (IL-10, IL-12, and IFN-γ) (53). In this study, the levels of IL-12 and IFN-γ secreted by recombinant *Lactobacillus* 6aa-COE/*L. reuteri*-stimulated MoDCs were higher than those stimulated by IL-10, suggesting that CTLA4-6aa is beneficial to recombinant *Lactobacillus* to stimulate DCs to mediate the cellular immune response and differentiate T-cells into Th1 type cells.

An effective oral vaccine can induce both systemic and mucosal immune responses. Therefore, we evaluated the immune response of piglets induced by recombinant *Lactobacillus via* oral immunization. Serum-derived IgG can improve the body’s immune defense by reducing pathogens’ ability to cross the intestinal mucosa, thereby effectively blocking the systemic transmission of pathogens to the organism (54). sIgA plays an important role in the intestinal mucosal immune response and is a major immunoglobulin in mucosal immune responses that protects against pathogen invasion at mucosal sites (55). In this study, we detected high levels of antigen-specific mucosal sIgA in the feces, nasal cavity, and serum of piglets orally immunized with 6aa-COE/*L. reuteri* and anti-PEDV-specific IgG and sIgA levels significantly increased in the 6aa-COE/*L reuteri* group after immunization than the PBS and the recombinant *Lactobacillus* COE/*L. reuteri* group. The SIgA and IgG contents tended to increase with time. The level of SIgA began to decrease on the twenty-first day after immunization, but the IgG from the blood showed a downward trend on the thirty-fifth day after immunization, indicating that the mucosal immunity induced by recombinant *Lactobacillus* occurred earlier than humoral immunity. Still, the humoral immunity produced lasted longer, which was confirmed in our previous studies (56, 57). In the present study, we demonstrated oral vaccination with recombinant *Lactobacillus* 6aa-COE/*L. reuteri* strongly induces systemic and mucosal immune responses against PEDV. These results indicated that recombinant *Lactobacilli* expressing CTLA4-6aa have a strong ability to promote DCs maturation and initiate immune responses.

Taken together, this study confirmed that recombinant *Lactobacillus* expressing CTLA4-6aa was more efficiently captured by porcine DCs and had an increased ability to promote DCs maturation. Additionally, we confirmed that the LYPPPY motif of porcine CTLA4 is the key sequence for its interaction with B7 in porcine DCs. Our results showed that antigen targeting of DCs using *Lactobacillus is* a promising strategy for the development of porcine vaccines.

## Data availability statement

The original contributions presented in the study are included in the article/**Supplementary Material**. Further inquiries can be directed to the corresponding authors.

## Ethics statement

The animal study was reviewed and approved by the committee on the Ethics of Animal Experiments of Northeast Agricultural University, Harbin, China.

## Author contributions

TX and NW designed and conducted research experiments. TX and NW analyzed data and wrote the paper. YT, CG, and JH contributed to isolation and validation of porcine MoDCs. YG, XW, JL, HZ, WC, ZS, YJ, XQ, LT, LW and YL performed research. All authors contributed to manuscript revision, read, and approved the submitted version.

## Funding

This work was supported by grants from the National Natural Science Foundation of China (Nos. 31772779 and 31972718).

## Acknowledgments

We would like to thank Editage (https://www.editage.cn/) for English language editing.

## Conflict of interest

The authors declare that the research was conducted in the absence of any commercial or financial relationships that could be construed as a potential conflict of interest.

## Publisher’s note

All claims expressed in this article are solely those of the authors and do not necessarily represent those of their affiliated organizations, or those of the publisher, the editors and the reviewers. Any product that may be evaluated in this article, or claim that may be made by its manufacturer, is not guaranteed or endorsed by the publisher.

## Supplementary material

Figure S1 Schematic diagram showing the construction of recombinant plasmids. ①6aa-COE fragment, cleaved with SacI and ApaI, was inserted into constitutive expression vector pPG-T7g10-PPT, and recombinant plasmid pPG-T7g10-6aa- COE was obtained by blunt end ligation.② 6aa gene in pPG-T7g10-6aa-COE was disrupted by HindIII and XhoI, 87aa fragment, cleaved with HindIII and XhoI, was inserted into constitutive expression vector pPG-T7g10-PPT-COE, and recombinant plasmid pPG-T7g10-87aa- COE was obtained by blunt end ligation.

Figure S2 Morphology of peripheral blood mononuclear cells (PBMCs) cultured for 6 days in the presence of recombinant porcine granulocyte-macrophage colony-stimulating factor (GM-CSF) and interleukin (IL-4). Scale bar 100 µm **(A)**. Fluorescence microscope: FITC-labeled MHC class II is shown in green. Phycoerythrin-labeled CD172a is shown in red. The 4’,6-diamidino-2-phenylindole stained nucleus is shown in blue. Scale bar = 100 µm **(B)**. Flow cytometry: counts of FITC-labeled MHC class II and phycoerythrin-labeled CD172a expressed on immature porcine monocyte derived dendritic cells (MoDCs), and the mean fluorescence intensities of FITC-labeled MHC class II and phycoerythrin-labeled CD172a expressed on immature porcine MoDCs **(C)**. Different letters (a vs. b, a vs. c, b vs. c) indicate significant differences (*p* < 0.01) at the same time point.
