# Supplementary figures and images for: Delivery of antigen to porcine dendritic cells by fusing antigen with porcine dendritic cells targeting peptide
Source: Front Immunol. 2022 Sep 8;13:926279. doi: 10.3389/fimmu.2022.926279 (PMC9499840; doi:10.3389/fimmu.2022.926279)

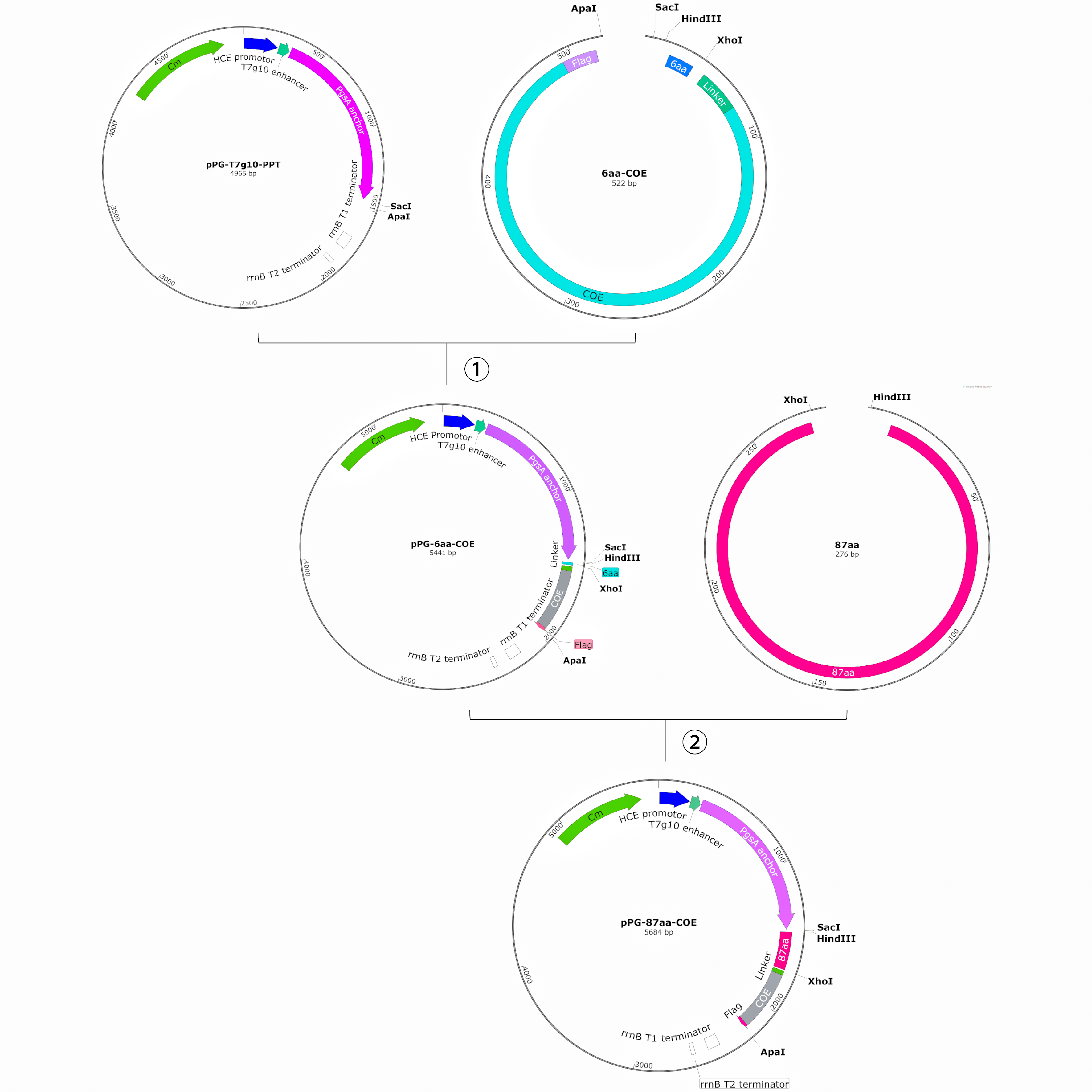

Supplement: Figure S1 — Schematic diagram showing the construction of recombinant plasmids. ①6aa-COE fragment, cleaved with SacI and ApaI, was inserted into constitutive expression vector pPG-T7g10-PPT, and recombinant plasmid pPG-T7g10-6aa- COE was obtained by blunt end ligation.② 6aa gene in pPG-T7g10-6aa-COE was disrupted by HindIII and XhoI, 87aa fragment, cleaved with HindIII and XhoI, was inserted into constitutive expression vector pPG-T7g10-PPT-COE, and recombinant plasmid pPG-T7g10-87aa- COE was obtained by blunt end ligation. [file Image_1.tif]

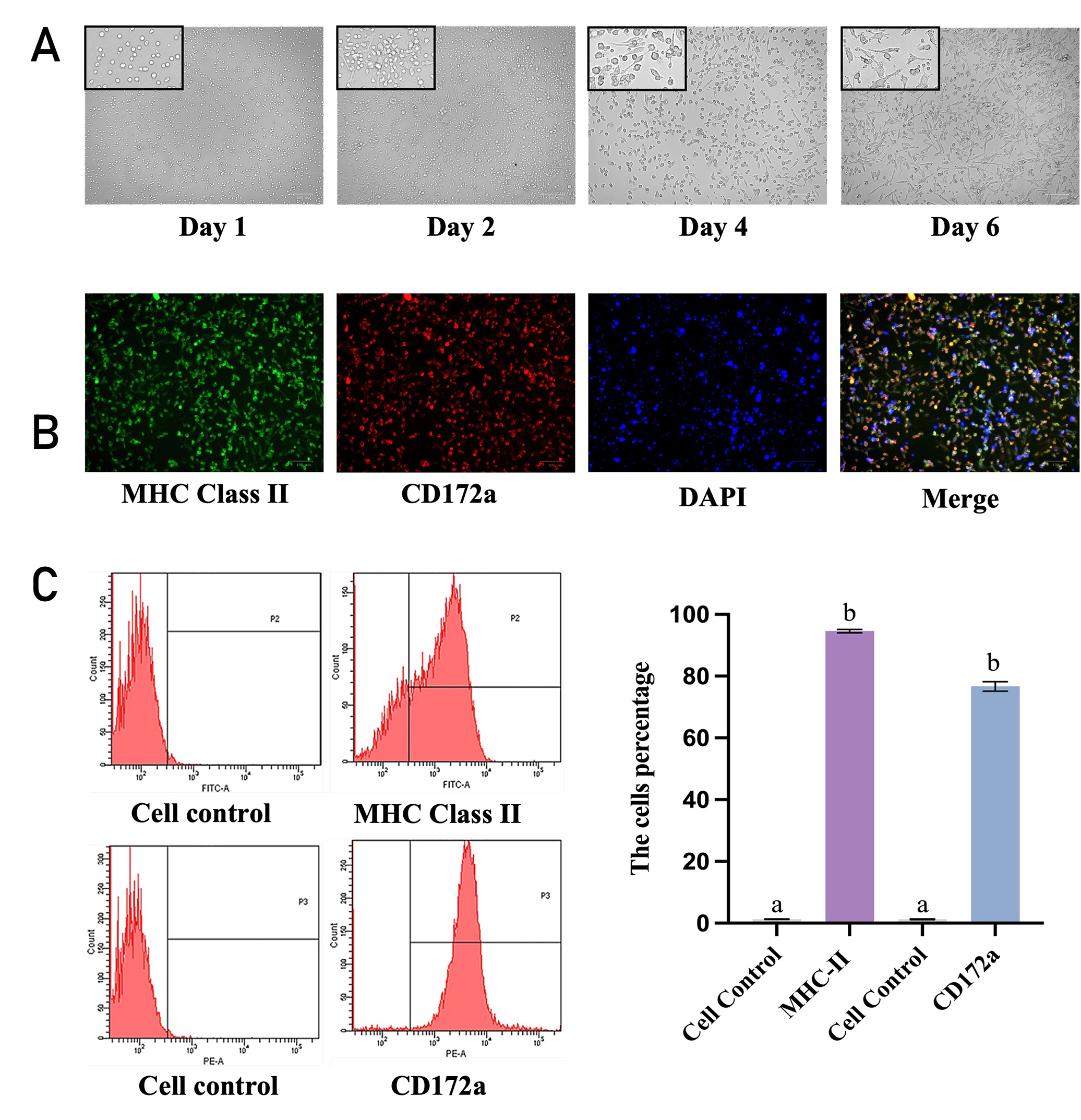

Supplement: Figure S2 — Morphology of peripheral blood mononuclear cells (PBMCs) cultured for 6 days in the presence of recombinant porcine granulocyte-macrophage colony-stimulating factor (GM-CSF) and interleukin (IL-4). Scale bar 100 µm (A). Fluorescence microscope: FITC-labeled MHC class II is shown in green. Phycoerythrin-labeled CD172a is shown in red. The 4’,6-diamidino-2-phenylindole stained nucleus is shown in blue. Scale bar = 100 µm (B). Flow cytometry: counts of FITC-labeled MHC class II and phycoerythrin-labeled CD172a expressed on immature porcine monocyte derived dendritic cells (MoDCs), and the mean fluorescence intensities of FITC-labeled MHC class II and phycoerythrin-labeled CD172a expressed on immature porcine MoDCs (C). Different letters (a vs. b, a vs. c, b vs. c) indicate significant differences (p < 0.01) at the same time point. [file Image_2.tif]
